# Comparative neuroanatomy of emotion expression: from flies to humans

**DOI:** 10.1098/rstb.2025.0135

**Published:** 2026-09-24

**Authors:** Ralph Adolphs

**Affiliations:** Department of Biology and Biological Engineering, California Institute of Technology, Pasadena, CA 91125, USA

**Keywords:** emotion, comparative physiology, facial expression, hypothalamus, amygdala, periaqueductal grey

## Abstract

All animals exhibit complex behaviours that share features with those that in humans we call ‘emotional expressions’. Sidestepping contentious issues about the conscious experience of emotion, their expression across species provides a wealth of detail about their emergence in evolution, the shared features that distinguish them from reflexes, and principles for their neural control. This paper provides a high-level survey of this vast field.

This article is part of the theme issue ‘Mechanisms, development, phylogeny and functions of emotional expressions’.

## 1. Introduction

Behaviours caused by emotional states are ubiquitous across species, both vertebrate and invertebrate [1]. For example, an octopus will inflate itself when faced with a threat, a behaviour analogous to piloerection in mammals. If the threat is imminent and severe, the octopus will propel itself away and ink, analogous to a mouse darting into its burrow. If the threat is not imminent and merely anticipated, the octopus may camouflage itself, just as a mouse may freeze.

As these brief examples illustrate, the clearest emotions across species are probably those related to defensive and aggressive functions. Arguably, mating and parental behaviours, as well as play [2], can be added as positively valenced examples; but here the details vary tremendously, making broad cross-species comparison less useful for extracting shared functional themes and design principles. Essentially, all animals face predation (even the young of apex predators), and all need to compete for resources within a species. These shared themes impose functional constraints that have sculpted the evolution of neural circuits responsible for their implementation.

Two important preliminary issues need to be addressed first. One issue is that emotions can modulate everything, so we need to constrain the topic of ‘emotion expression’. The second is that we need to clarify what we mean by ‘emotion’. With respect to the first issue, it is an ingredient of essentially every emotion theory that emotions coordinate widespread effects on cognition and behaviour. The literature on this topic is vast. In humans, there are well-known effects of emotion on memory, attention and decision-making [3]. In other animals, similar effects have been documented, extending to ‘cognitive biases’ in decision-making even in bees when agitated [4]. Emotions will bias behavioural choices, reveal themselves in goal-directed behaviour and modulate ongoing movement (see Braine & Georges [5] for a detailed review in humans and mice). Many of these effects of emotion, however, are not the direct, high-priority behaviours that are most rapidly triggered by an emotion, and in many cases they are not causal effects of what we would properly call an emotion at all (but of a different type of affective state or mood). We will focus here on direct, rapid, relatively automatic and stereotyped emotional behaviours like fighting and fleeing, not on the more distributed effects on goal-directed behaviour or effects on decision-making or other cognitive processes that may ultimately also bias behaviour.

**Figure 1.**
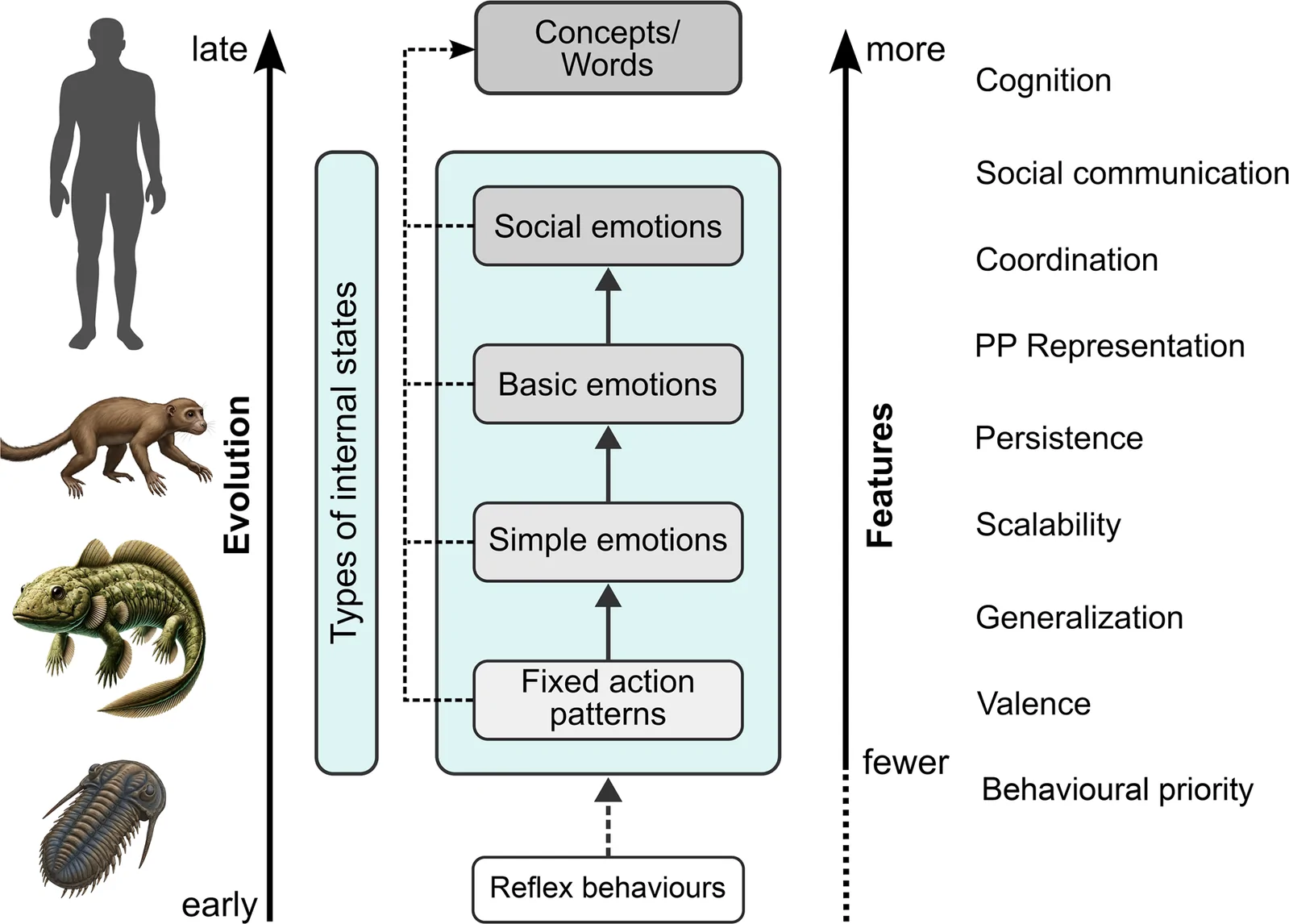
The evolutionary elaboration of emotions and their features. A conceptual schematic that outlines the evolution of emotions and their differentiation across species. The precise set of features shown on the right, and the order in which they were added, remains provisional. The features are also not all of the same type—some are properties of the emotion state itself, some are properties of the way the emotion is used by a mechanism to cause behaviour, and some are psychofunctional features. As the features come together in a package, they characterize emotions from simple to more complex, as suggested in the figure. An important point is that the species shown on the left are intended to show ancestors along the evolutionary line to humans, which are not species we can study today. From [10]. PP Representation: pushmi-pullyou representation, a type of representation in philosophy of mind that represents both the situation causing the emotion (what the emotion is about) and actions to be taken (what the emotion motivates). The first type of representation can be thought of as a map, the second as a blueprint.

The second issue is equally important. It is sometimes thought that not all species can display emotional behaviours, since not all species can have emotions. For instance, whether or not insects have emotions is a contentious question. But the reason that it is contentious is easy to identify: many, perhaps most, approaches to emotions conflate emotions with feelings (that is, with conscious experiences of emotions) and we don't have any clear criteria for inferring whether animals have conscious experiences (of emotions or of anything else) [6]. If we adopt a framework that is agnostic about whether or not an emotion might also be accompanied by a conscious experience of that emotion, we can come up with criteria for attributing emotions—perhaps in a more primitive form—across different species [7,8]. This approach of investigating emotions across species, conceiving of emotions as functional states that are conceptually independent of conscious experience, is also the one adopted in this paper. Importantly, it does not at all follow from this that therefore we have decided that animals do not have conscious experiences of emotion. We have just separated that very difficult question from the more tractable question of whether they have emotional states. According to one recent framework outlined in [7] and [9], we can list some functional criteria for attributing emotions to other species, without needing to worry about whether they consciously experience them. A provisional list of those criteria is reproduced in figure 1, and we will return to it repeatedly throughout this paper.

The list of emotion features shown in figure 1 was generated from two considerations. First and most simply, it is the list of features that are shared in common by emotions about which there is no disagreement—defensive and aggressive states, which have been the best studied, as well as disgust and social emotions, all share most or all of these features. Second, and more usefully, the features are ones that one would derive—and engineer into an artificial system—in order for emotions to carry out their function as evolved states that control behaviour at a level that is intermediate to reflexes (which are too narrow and rigid) and volitional goal-directed behaviour (which is too slow). In my view, emotions evolved when brains were able to abstract statistical regularities shared between thematically related environmental challenges [10].

It is also useful to further circumscribe what we mean by emotions. To some extent, this is just a semantic issue—we can label these categories as we like, but I think it is useful to make distinctions. Thus, emotions triggered by highly survival-relevant external stimuli (like predators) are the category on which this paper focuses. Those emotional states should meet most of the criteria listed in figure 1. There are many other kinds of internal states that also share some of these criteria with emotions, but they differ from emotions because they are not about a stimulus in the environment (e.g. hunger, thirst, pain and other interoceptive and homeostatic states), or because they are not sufficiently differentiated (e.g. startle and pain), or because they are not phasic (moods). Emotions have strong effects on both striate and smooth muscles, in addition to their widespread endocrine and cognitive components. Here, we will focus on striate muscle effects that correspond to visible overt behaviour. Three broad categories of emotional behaviours can be distinguished here: vocal, facial and movement. Of these, facial expressions have been studied the best in primates, whereas movement has been studied the best in other species.

## 2. Subcortical emotion circuits in vertebrates

**Figure 2.**
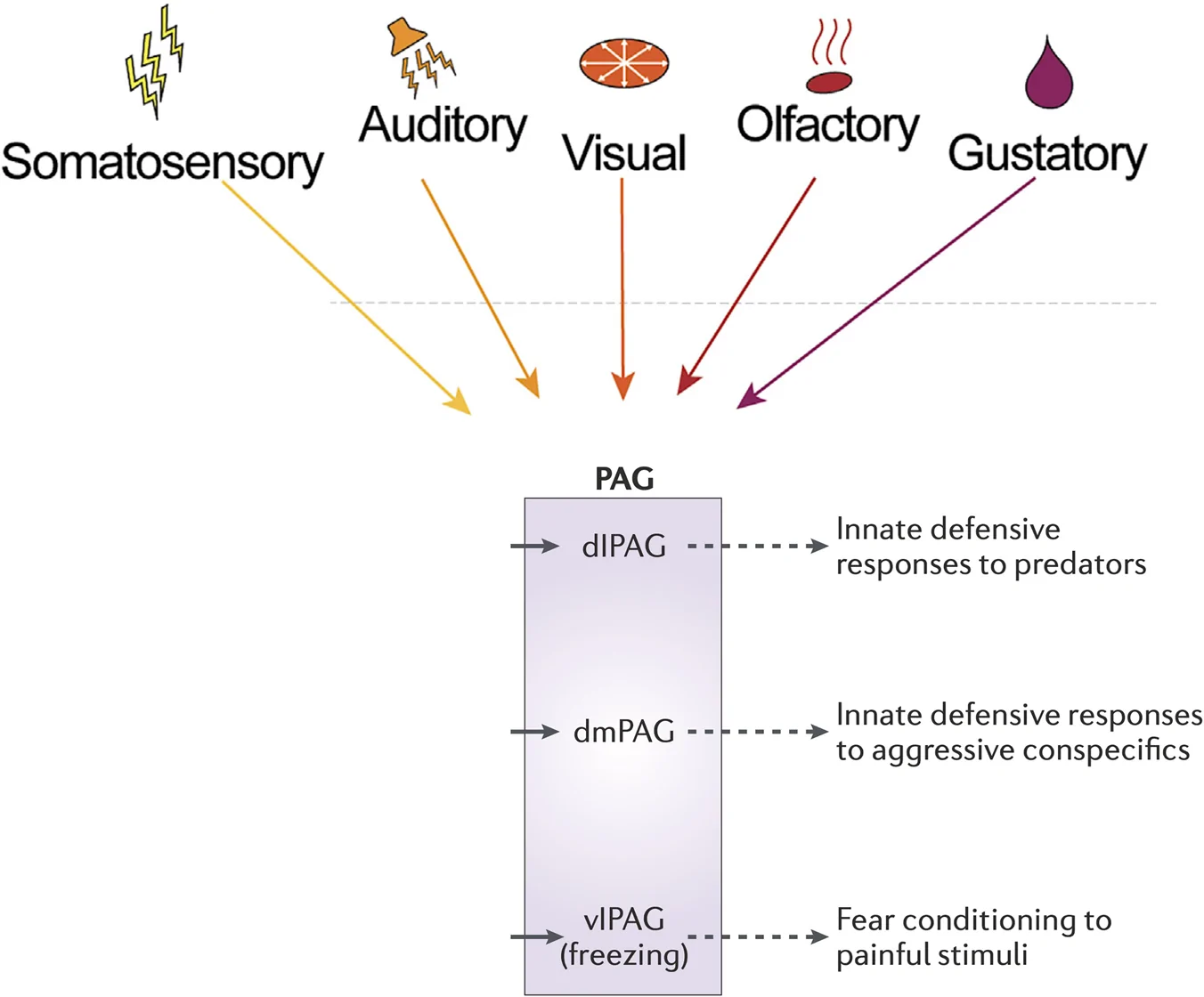
The periaqueductal grey (PAG) implements a ‘fan-in/fan-out’ architecture for emotion expression. A schematic example is shown from the PAG circuits in rodents, but the basic scheme applies broadly across vertebrate species. Images modified from [11] and [12]. dlPAG, dorsolateral PAG; dmPAG, dorsomedial PAG; vlPAG, ventrolateral PAG.

Choosing a more central focus than facial expression or vocal control, there are conserved subcortical nuclei in vertebrates that coordinate all the disparate causal effects of an emotion (including autonomic effects that we are not discussing here). Very roughly, one can think of a set of appraisal-related circuits that consist of rapid sensory processing, often involving specific ‘labelled-line’ channels, which feed into a set of subcortical nuclei encompassing amygdala, hypothalamus and parabrachial nucleus, as well as some thalamic nuclei. This ‘fan-in’ architecture generally provides central emotion systems with multi-sensory, abstracted information about salient environmental challenges—like predators, prey, mates or conspecific competitors. A final funnelling of this processing prominently involves a large and complex subcortical structure: the periaqueductal grey (PAG), which in turn orchestrates the main diverse downstream effects of an emotion, including its overt expression (a ‘fan-out’ architecture). Thinking of the flow from sensory input to motor output in this ‘fan-in/fan-out’ fashion positions the PAG at the bottleneck (figure 2).

By far the best studied systems for emotion expression in terms of detailed neural circuits derive from a set of four subcortical nuclei in rodents: the superior colliculus, the amygdala, the hypothalamus and the PAG. Whereas the PAG can, to some extent, be considered as coordinating the multi-component emotional behaviour (although it has complex feedback projections to all other regions as well), the other three nuclei have more specific functions in the control of particular types of emotions.

Perhaps the most modular here is the superior colliculus, which constructs an abstract sensory representation of threat (from both auditory and visual inputs). Noisy population-wide sensory evidence accumulation in cells in the superior colliculus tracks predator imminence: the signal builds up with visual looming stimuli or auditory stimuli of increasing amplitude. These sensory features reflect the approaching shadow of an aerial predator, like a hawk, or alarm calls of conspecifics, respectively. This abstract threat representation is then read out by cells in the PAG, which implement a thresholded decision of a mouse to freeze or run away, depending on context (proximity to a safe hiding place) [13]. The elegance of this circuit notwithstanding, it is of course not completely encapsulated—there are connections with other structures like the thalamus, and there is even the requirement for input from the primary visual cortex to gate responses in the superior colliculus.

Although also long considered a module for fear of sorts, the function of the amygdala is much more complex. While visual response latencies in the superior colliculus are generally 40–60 ms, those in the primate amygdala are typically 100–300 ms. The reasons for this longer latency reflect the complexity just alluded to: whereas the superior colliculus receives direct retinal input, the amygdala receives only highly processed visual input, predominantly from anterior temporal cortex (there is continuing debate about a more rapid route via the pulvinar thalamus, but in any case, this appears to be a tiny contribution to the amygdala's neural responses; [14]). Furthermore, there is massive contextual input from the frontal cortex, making it likely that the amygdala is multiplexing signals from many sensory modalities, together with contextual and goal-related information in order to represent emotion [15].

The PAG is the most relevant structure here in terms of neural control of emotional expression. It funnels multisensory information to a number of downstream targets in the medulla that, in turn, project to motor neurons as well as autonomic nuclei and ganglia to coordinate the many components of emotional expression. These encompass changes in breathing, blood pressure, heart rate and essentially all sympathetic and parasympathetic autonomic targets, as well as complex sequenced behaviours (e.g. freezing or fleeing). The behaviours orchestrated by the PAG were first mapped out by injecting excitatory neurotransmitters directly into the brains of rodents, revealing a columnar structure of PAG neurons that surround the central canal [16]. Whereas the lateral PAG is more involved in defensive behaviours together with accelerated heart rate and increased blood pressure, the ventrolateral PAG is associated with freezing and hiding, together with decreased heart rate and decreased blood pressure. Modern studies using optogenetics with cell-type specificity have further fractionated different PAG regions, but the general organizing principle of a columnar organization that maps functionally relevant emotional expressions has remained. There is domain-specificity as well: while the dorsolateral PAG is associated with defensive responses to predators, the dorsomedial PAG is associated with defensive responses to conspecifics (figure 2).

Two further considerations of the PAG are informative for how we think about emotional expression. One critical note is that many of the behaviours caused by the PAG are, in fact, inhibitions of behaviour. Initial interruption of ongoing behaviour and alerting would be one example, rigid freezing with strong inhibition of all visible action would be another. These inhibitory aspects of motor control are as important as those that trigger visible sequences of behaviour [17]. A second consideration is the emergence of the PAG as a main controller and coordinator of emotional expression in evolution. Given the PAG's ubiquitous role in essentially all homeostatic regulation, from breathing to cardiovascular control to responses to pain and inflammation, it seems likely that its coordination of the behaviours seen in emotion expressions co-opted these building blocks. While many of the functions of the PAG have been established from work in rodents, a similar evolution seems to have taken place in the assembly of the complex behaviours that constitute bird courtship behaviours, for instance [18]. The assembly of new and often more complex behaviours from pre-existing building blocks is a general feature of evolution across species, and the evolution of emotion is no exception here.

Appetitive behaviours associated with group cohesion, parental behaviours and mating involve some of the same subcortical brain structures, as well as distinct circuits. The classic reward pathway that probably exists in most vertebrates, from the ventral tegmental area to the nucleus accumbens, is thought to play a role ubiquitously in appetitively valenced behaviours. These structures are components of a distributed network within which stimulation can trigger positively valenced behaviours. Moreover, there is some evidence from work in rodents, both in subcortical structures like the nucleus accumbens as well as in cortical structures like the orbitofrontal cortex, that there may be a topography for valence, with appetitive and aversive behaviours triggered by anatomically segregated subregions [19]. An intriguing synthesis of positively valenced states in birds has hypothesized that singing is motivated by a system representing reward anticipation, involving the preoptic hypothalamus, ventral tegmental area and nucleus accumbens [20].

While the classic reward system whose core structures include the ventral tegmental area and nucleus accumbens has been by far the best studied with respect to appetitive behaviours, it is unclear whether this system should be considered an emotion. Its very broad generalizability instead serves a fundamental role in motivation and learning—a mechanism critical for remembering how best to get to foods, but perhaps better considered a broad type of motivational system than a type of emotion system. The behaviours evoked can be extremely diverse, unlike the more stereotyped behaviours generally associated with emotions.

This does not mean that the reward system has no part in positively valenced emotions, but it plays a role primarily in learning to associate new cues with unconditioned positive emotions. Those unconditioned positive emotions are rather less differentiated than negatively valenced emotions—perhaps Tolstoy was right when he noted that all happy families are alike, whereas all unhappy ones are unhappy in their own way. The core positive emotions seem to be mating and play. I would not consider hunger or thirst an emotion, although the consumptive behaviours that satisfy these motivational states show some features shared with emotions. It is interesting to note that mating and play depend on neural populations in close proximity to those mediating aggressive behaviour, in the hypothalamus and PAG, respectively [2,7]. This scheme requires mutual inhibition between the corresponding cell populations and complex regulation to avoid switching from a positively valenced behaviour to a negatively valenced one.

## 3. Emotion circuits in invertebrates: from command neurons to central states

Invertebrates provide a wealth of information about emotions and offer excellent model systems [21]. The dissection of escape behaviours in crayfish, squid and other invertebrates has famously demonstrated that they arise from very specific neural circuits that are thought to be both necessary and sufficient for the behaviours. Necessity is typically demonstrated by showing that lesioning the neural circuit precludes the behaviour; and sufficiency is established by showing that artificial stimulation (e.g. by electrical or optogeneticontogenetic stimulation) causes the behaviour, but both conclusions also require various further assumptions (e.g. stimulation also requires intact other components). Some analogous modules have also been found in vertebrates, for instance, in the Mauthner cells of goldfish. In all these cases, there is a small set of neurons (or even a single one) that deterministically causes the behaviour, generally with reflex-like rapidity and rigidity. The behaviours, moreover, are all-or-none and relatively tightly linked to the eliciting stimulus. These so-called ‘command-neuron’ circuits [22] are found ubiquitously across species, but clearly lack key features of emotions. They are generally not scalable but binary (off or on), they are generally not very persistent but phasic, and they do not abstract over either sensory inputs or behavioural outputs (they lack ‘fan-in/fan-out’ architecture).

Relatively reflex-like circuits are also seen for some emotion-like behaviours in simple vertebrates. For instance, the larval zebrafish hunts single-celled animals, a behaviour showing coordination and persistence as in the case of emotions. Yet, the circuits for triggering this behaviour are too simple to qualify as realizing an emotion. They involve rather rigid detection of specific visual motion cues in the prey (albeit probably involving the accumulation of evidence to a decision threshold) and a fairly shallow computation that culminates in the reticulospinal system for producing bouts of swimming behaviour. Like the command neurons in invertebrates, sensorimotor systems for these behaviours are better thought of as what the ethologists had called fixed action patterns—they lack the ‘fan-in/fan-out’ architecture characteristic of emotions, and consequently they are incapable of constructing central representations that abstract over the stimuli. Related types of behaviour in vertebrates may be the mating display behaviours of birds in courtship: they are more complex than reflexes, but less flexible than emotions proper.

Another interesting reflexive class of behaviours that are related to emotional expressions are deimatic behaviours: the stereotyped defensive pose adopted by many animals in response to undifferentiated threat (see [23], for review). This is a heterogeneous class of behaviours that, depending on how we choose to classify them, may even include primitive emotions. They include the stereotypical behaviours of many animals to make themselves appear larger, to camouflage themselves, or to mimic dangerous or poisonous stimuli, in an effort to ward off potential predators. The neural circuits are generally, again, relatively narrow, lacking a ‘fan-in/fan-out’ scheme: a reflex-like sensory trigger is channelled to a specific neural circuit that rigidly causes the behaviour.

**Figure 3.**
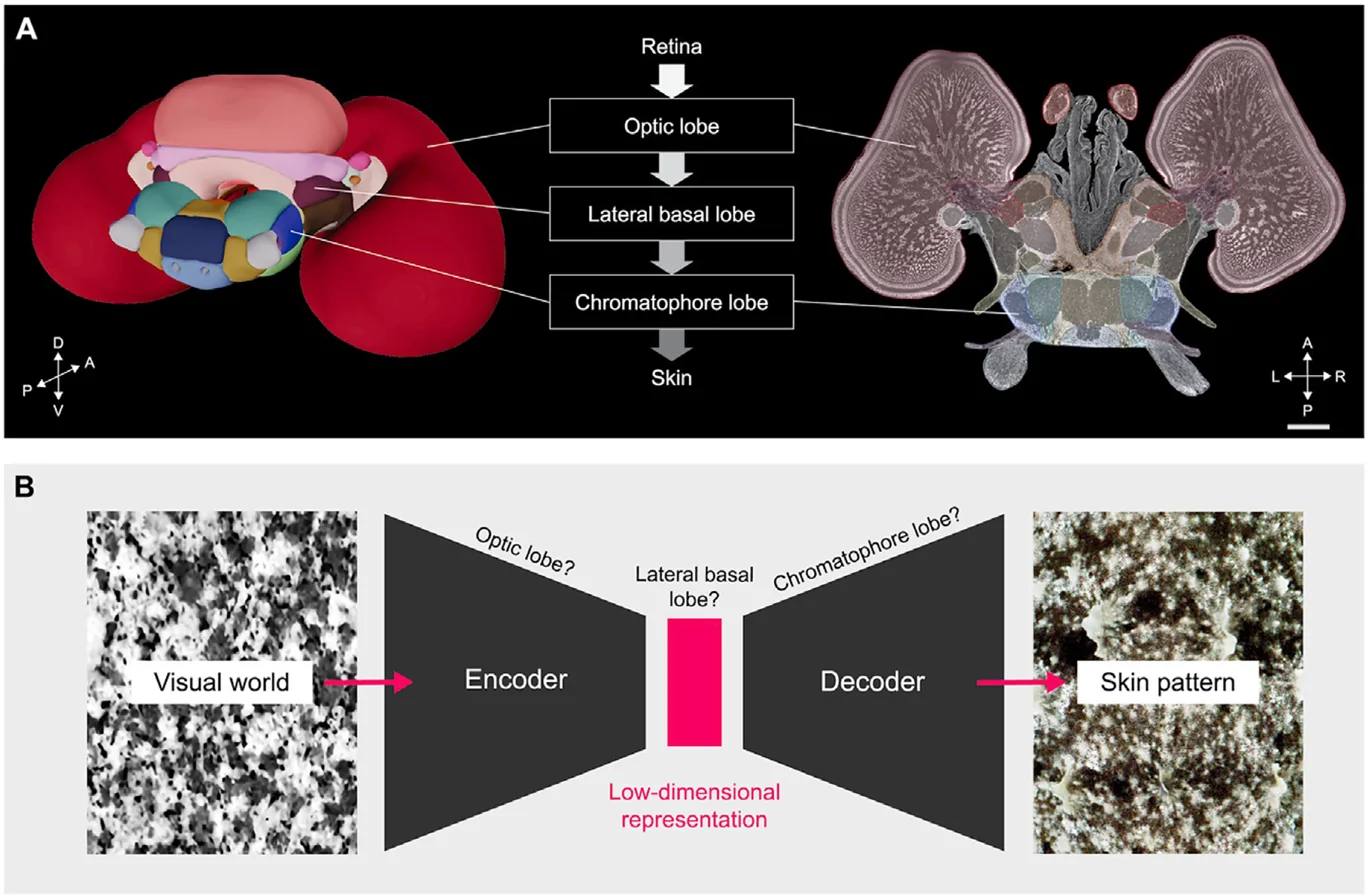
Neural example of the ‘fan-in/fan-out’ architecture of emotions. Shown is the example of camouflage behaviour in the octopus, although the conceptual point applies broadly across species. High-dimensional sensory inputs are compressed to an abstract central emotion representation (e.g. of threat), which is in turn assembled to a high-dimensional coordinated multivariate emotional expression. Extracted from [25].

Yet some emotional behaviours that can be described as deimatic, as well as others related to stalking prey by camouflage, begin to show many of the properties of emotions: they are highly context-sensitive, under dynamic and flexible neural control, and feature substantial central processing. Some of the most intriguing examples come from the complex colours and textures that can be displayed on the body surface of cephalopods (cuttlefish, squid and octopuses). These animals have by far the largest invertebrate nervous systems, rivalling the total cell counts of a ferret (ca 500 million neurons altogether in a large octopus), although they are arranged in alien constellations of ganglia and a substantial portion is distributed throughout the body. Although motor control in an octopus is decentralized in profound ways (largely owing to the high degrees of freedom in moving the arms; [24]), neural control of the skin shows a hierarchical control. High-dimensional visual information is compressed to lower-dimensional representations of the statistics of the environment, which in turn triggers high-dimensional control of the millions of chromatophores on the body surface. We see here parallels to the ‘fan-in/fan-out’ architecture of emotion processing that is a hallmark in vertebrates, pointing us to some profound shared principles (figure 3). A fascinating aspect of the high-dimensional control of body pigmentation in these animals is the possibility of obtaining a much higher fidelity read-out of an internal state. Whereas humans typically produce very coarse and categorical reports of their emotions in most experiments (verbal labels, like ‘afraid’), cephalopods could show us nuanced shades of many more emotion states from the dynamic patterns on their body surface (cf. [26]).

The class of emotional behaviours that have perhaps been best studied are aggressive behaviours towards conspecifics. One simple reason for this is that such behaviours are often sexually dimorphic and under the control of neural circuits or hormones that differ between the sexes. Consequently, one can identify the neural circuits and manipulate their activity by capitalizing on this dimorphism (e.g. searching for cells expressing hormone receptors or constructing molecular biology tools that target cells expressing the genes for such receptors). The vinegar fly, *Drosophila*, long a workhorse for all kinds of manipulations through molecular biology tools, shows sex-specific aggressive behaviours (in both sexes) whose control has been traced to a small number of central neurons that function in many respects like command neurons [27]. Given that we now also have the *Drosophila* connectome available, further analyses are available to fully understand the downstream effects of these neurons. As with other stereotyped behaviours in flies (e.g. grooming), the neurons do not by themselves cause the behaviour, but they recruit additional neurons within a network first and function more to change the likelihood with which external stimuli can evoke emotional behaviours. This conclusion probably also holds for optogenetic manipulation of central circuits for aggression in vertebrates.

The scalability and persistence of internal emotion states that are also seen in invertebrates give us some clues to how this might be biologically realized. So far, two main implementations seem apparent. One involves recurrent neural activity, so that feedback loops and features of dynamical systems can exhibit long-lasting states that could not be achieved by simple feedforward neural circuits. Such recurrent neural activity, based on recent elucidation of the *Drosophila* connectome, has been proposed to underlie persistent states for mating and aggression in flies [28]. A second implementation instead relies on slow-acting and persistent neurotransmitters, notably neuropeptides. It has long been known that biogenic amines like serotonin or peptides like tachykinin increase aggressive behaviour in several animal species, from lobsters through flies. Neuropeptides are also prominently involved in vertebrate emotions; for instance, cholecystokinin administration can cause panic attacks even in humans. While in many cases, neuropeptides may simply induce neural activity relevant to an emotional state, whose persistence could then be maintained by recurrent connections, there is recent evidence in rodents that neuropeptides (oxytocin and vasopressin, in that study) can also function to implement temporal persistence within the relevant neuronal populations [29].

## 4. Facial expressions

By far the most intensively researched domain of emotional expression is human facial expressions. These are the predominant non-verbal channels for social communication in humans, have historically been associated with specific emotions and are distinguished in their complexity from other channels. The late Paul Ekman and colleagues first devised the Facial Affect Coding System (FACS), which decomposes configurations of facial expressions to 44 objective combinations of specific muscles [30]. Ascribing these to specific (discrete) emotions requires a further step, with complicated questions about validity [31]. The FACS system has by now been extended to dogs, cats, horses, chimpanzees, orangutans, gibbons, macaques and marmosets [32] and research using FACS continues to be actively pursued (including using it as a basis for emotion classification for machine vision/AI algorithms).

An interesting study used machine learning to classify putative emotions even from the facial movements of mice [33]. That study also illustrates many of the challenges noted above: among the emotions classified were pain, bitter and sweet taste. It is not obvious that these are emotions, although they clearly are internal states that share some functional properties with emotions (and involve emotions in various ways). Nonetheless, this particular study is a very important example of how new emotions could be discovered even in animals quite different from humans, whose behaviour we might otherwise tend to anthropomorphize. In the study by Dolensek *et al.* [33], unsupervised machine vision methods were used, rather than the FACS coding available for larger animals. The movements of the mouse's face and head were captured with high-resolution video, and these raw data were then clustered into categories that could be related to the situations that evoked them. Interestingly, several of the features of emotions that we listed in figure 1 emerged from this analysis: the study found evidence for intensity, persistence and generalization. The study furthermore found a correlation between these behavioural categories based on facial movements, and patterns of neuronal activation in an interoceptive cortex that probably represents the sensory consequences of those facial actions—the insula.

Exactly how the facial muscles function to warp the connective tissues and skin and thereby result in visible facial movements is complex—generally, one end of the muscle is inserted into the bone, whereas the other end serves to pull tissue. However, individual facial muscles are heterogeneous, both in their size and types as determined by staining—a complexity that probably also contributes to individual differences in how facial movements can be made. Another odd feature of facial muscles, at least in humans, is that a single muscle fibre can be innervated by multiple axons, a feature normally seen only early in development, which complicates a simple account of how facial expressions are controlled neurally.

In primates, the facial muscles are under complex neural control, innervated by multiple distinct axons originating from a brainstem nucleus, the facial nucleus, which is the largest brainstem motor nucleus in humans and a nucleus that is proportionately larger in apes and humans than in monkeys, probably reflecting the more extensive use of facial expressions in social communication in those species. Descending inputs to the facial nucleus, in turn, originate from multiple cortical and subcortical regions, some of which are generally ascribed ‘emotional’ and involuntary control (in humans), whereas others offer volitional control. Distinct subdivisions of the facial nucleus innervate different regions of the face, a topography that varies with the use of specific facial muscles across species. For instance, vibrissae use depends on projections from the lateral facial nucleus, whereas the intermediate and dorsal nuclei play more of a role in the muscles responsible for facial expressions in primates. Each facial nerve, on the left and right, innervates the muscles on only that half of the face. There also appears to be crosstalk between the sensory trigeminal nerve and the facial nerve, but the functional significance of this remains quite unclear. In primates, the control of the upper and lower halves of the face is neurally distinct, with the consequence that the lower facial muscles are under considerably more voluntary control, whereas the upper facial muscles are more engaged during genuine emotions [34].

While there remains debate about any clear muscle somatotopy within the axons of the facial nerve, their cell bodies in the facial nucleus are organized in this way. Columns of facial motor neurons are arranged so that they innervate the same facial muscle, and different subdivisions of the facial nucleus innervate distinct regions of the face in a topographic fashion [35]. The simplest sources of control originate from a variety of reflex sources, including blinking or coughing, which involve multiple facial muscles. Many of these reflexes are also recruited during emotional expressions, emphasizing the complex loops by which facial movements unfold as dynamic processes. Unlike all other striated muscles, the facial muscles appear to lack a sensory proprioceptive component (i.e. muscle spindles) that would normally be a key component of such reflexes—but unlike other muscles, the facial muscles have no significant load for which to adjust. Nonetheless, the cutaneous sensory receptors in the skin of the face do convey information correlated with facial movements to the brain (see [36] for review).

**Figure 4.**
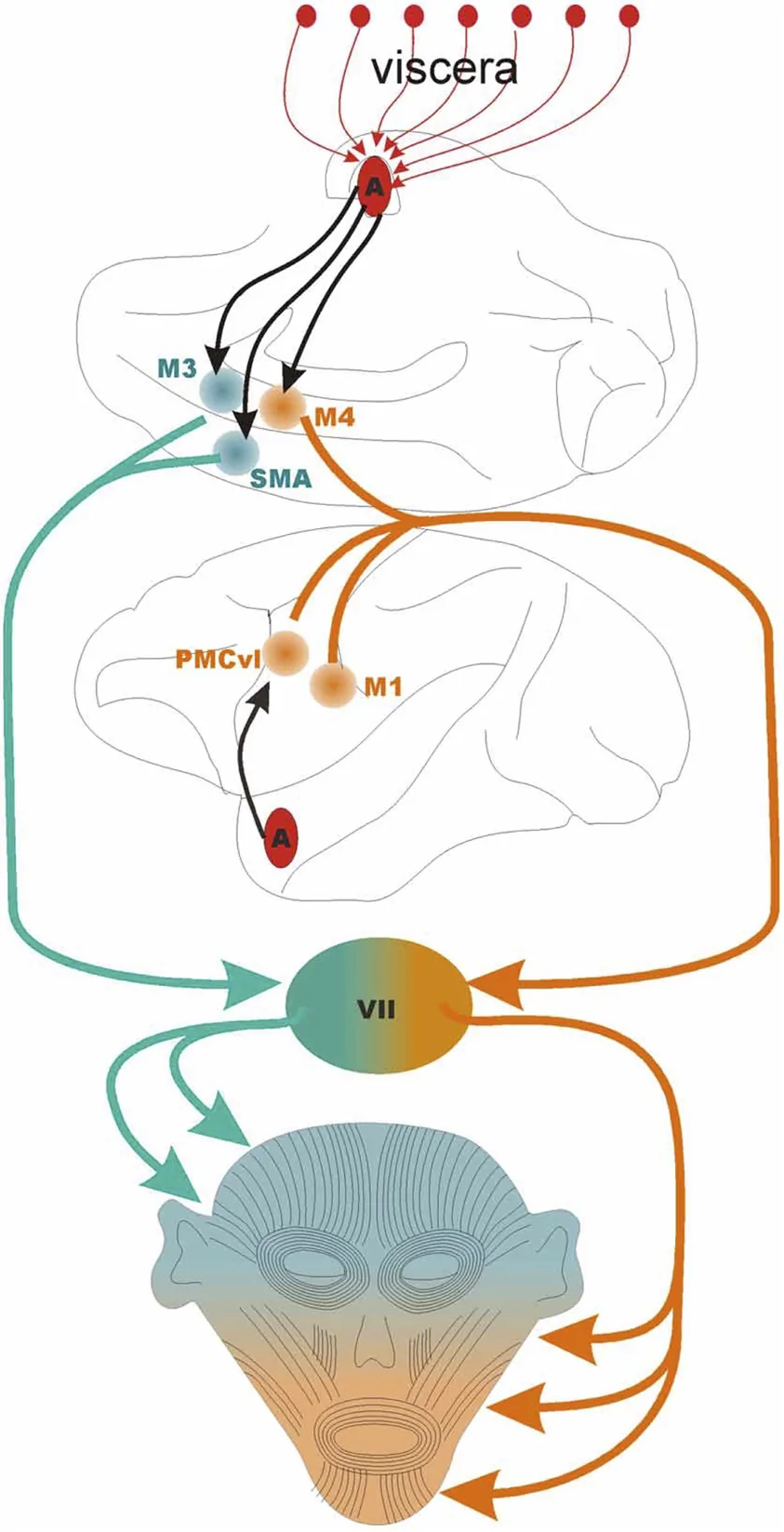
Descending control of the facial muscles in primates. The figure schematizes the topographic relationship between specific motor cortical regions, medial and lateral subdivisions of the facial nucleus, and the upper and lower muscles in the face. The cortical regions are innervated by the amygdala, which provides additional emotional control. From [34]. Abbreviations: M1, M3 and M4: motor cortices; SMA: supplementary motor area; PMCvl: cingulate motor cortex; A: amygdala; VII: facial nucleus.

Whereas descending inputs to the facial nucleus for voluntary facial movements come from primary motor and ventral premotor cortex (Bradman's areas 4 and 6), inputs for involuntary emotional expression originate from most of the subcortical structures we reviewed above (amygdala, hypothalamus and bed nucleus of the stria terminalis) that directly or (more commonly) indirectly converge on the facial motor nucleus. Importantly, this system for the emotional control of facial expressions is distinct from systems involving additional effectors, as in laughing or crying (although of course both can be engaged concurrently). Perhaps the most relevant cortical source of projections for emotional facial expressions comes from the cingulate motor cortex, which has direct projections to the facial nucleus, and in turn receives strong projections from the lateral basal amygdala that terminate mostly on the face representation in the cingulate motor cortex [37] (figure 4). The cingulate motor cortex is a phylogenetically older component of mammalian cortex specifically involved in regulating autonomic and somatic behaviours that are components of emotions. For instance, electrical stimulation of this region in monkeys can evoke emotional vocalizations, whereas stimulation in humans can cause laughter [38].

## 5. Enduring debates

There are perhaps three broad types of debates in emotion science on which we can briefly comment. The first we already noted at the outset: the confusion between emotion states, emotion concepts and emotion experiences. The second concerns the bounds of an emotional state anatomically: where does it begin and end in the brain? The third is a perennial argument among emotion theorists about whether emotions are discrete categories or dimensional. Each of these is informed to some extent by the neuroanatomical and neurophysiological data.

With respect to the first issue—the confusion between emotion states, concepts and experiences—there are interesting comparisons between animals and humans. Emotion states, the only type validly studied in animals, appear strongly dependent on the subcortical circuits that we discussed above—structures such as the amygdala, the hypothalamus, the superior colliculus and the PAG. While it is hard to know whether animals have emotional experiences, and unlikely that they have emotional concepts, it is possible to dissociate these in humans. The detailed study of a famous patient, S.M., who had complete bilateral amygdala lesions, provides a case study. This patient, just like animals with amygdala lesions, lacked many of the functional aspects of a fear state—she did not show Pavlovian fear conditioning [39] and exhibited behaviour indicating a lack of fear (e.g. handling of snakes and spiders; [40]). On the other hand, she was perfectly able to describe fear—including the kinds of stimuli that would cause fear and the kinds of behaviours that would be shown. She thus had a concept of fear—at least as far as could be expressed in language. In this respect, she had just what a large language model would also have. On the other hand, her conscious experience of fear seemed to be lacking, both in experimental situations and in the real world [41]. So, emotional states (and perhaps experiences) of fear depend on the amygdala; emotion concepts do not.

Conversely, emotion concepts and emotion experiences both recruit large regions of the neocortex when surveyed in careful neuroimaging studies in humans. For instance, one large-scale study presented participants with emotion-inducing films and then mapped the activation patterns in their brains as a function of the emotion features that had been annotated in those films [42]. Emotions that could be well predicted by the total fMRI activation pattern included ‘fear’, ‘anxiety’, ‘disgust’ and ‘amusement’, but also ‘calmness’ and ‘neutral’. Emotions that could not be predicted included ‘interest’, ‘nostalgia’, ‘guilt’, ‘boredom’ and ‘craving’. Although participants were rating their emotional experiences, these ratings were probably also contaminated by their emotional concepts—after all, they had to think about what they felt in order to give the ratings. Many cortical regions (and also subcortical regions) were involved, more in line with the relatively anatomically distributed view of constructed emotion theory [43]. But I think that the constructed emotion theory is not a theory of emotion states; it is a theory of emotion concepts. Emotion concepts in humans depend on the cortex; emotion states in humans and animals depend on subcortical structures.

Let us turn briefly to the second big question: where does an emotion begin and end? It is easy to eliminate some very early sensory processing (e.g. the retina is not a component of a central emotion state) and some very late motor processing (e.g. motor neurons in the spinal cord, or sympathetic chain ganglia, are not constitutive of a central emotion state). Instead, these structures serve as inputs to, and outputs from, an emotion state. Their relation to the emotion state is causal, not constitutive. To see the distinction, one could intervene on the structures and see whether that necessarily changes the emotion. I can be in a state of fear while dreaming or hallucinating, so the retina is not part of the emotion. I can be completely paralyzed or have locked-in syndrome and feel afraid, so the motor neurons are not part of the emotion.

Similar manipulations more centrally become more challenging to interpret. Is the PAG part of the central emotion state, or merely a kind of premotor structure for the expression of the emotion? Careful recordings and stimulation argue that it is part of the emotion state, together with other subcortical nuclei. The reason that the emotion state cannot be anatomically dissected beyond a point is simple: there are dense bidirectional connections between all of these nuclei. Instead, once we reach a sufficiently central position anatomically, we should treat the entire system of subcortical connected nuclei as the substrate for the emotional state and what distinguishes stages of emotion processing is now time rather than anatomy. Different kinds of models may be better suited for explaining emotions if we focus sufficiently on the central parts of the brain. Whereas a coarse perspective can give us anatomical distinctions between inputs and outputs (e.g. figure 2), the central components may best be modelled as coupled dynamical systems whose behaviour is characterized better by time than space (see below; [44]).

The third continuing debate concerns whether emotions are discrete or dimensional. I think this is a false dichotomy—they are both, depending on how you want to characterize them. There are certainly anatomical circuits for specific emotions, such as the subcortical ones we reviewed above. But all of those emotions share functional features (valence, scalability, persistence, etc.) that constitute a dimensional space onto which the emotions can be mapped. Consequently, every emotion can be related to every other emotion in that feature space—some emotions are more similar to one another than others. Those dimensionally defined similarities pertain to the abstract functional features of emotions (and perhaps are mirrored in emotion concepts and experiences). The discrete circuits pertain to the way the emotional states are physically realized in the brain.

## 6. Shared principles

The rapid expression of a highly coordinated, multi-component and temporally sequenced behaviour is a shared aspect of emotions across species. The ‘fan-in/fan-out’ architecture that enables this functional feature is also a shared aspect. However, both the multi-component aspect and the degree of coordination (the ‘fan-out’), as well as the degree of abstraction from the sensory properties of the stimulus (the ‘fan-in’) clearly vary across species and they tell us something about how emotions evolved. The rather stereotyped behaviours seen in insects, just like the mating rituals of birds, do not yet evidence the full flexibility of mammalian emotions because they lack some of the features depicted in figure 1—but they point us to more primitive building blocks out of which emotion expressions probably evolved (see [10] for a detailed discussion). These conclusions are speculative at this point—they suggest an agenda for accumulating considerably more evidence across species than is currently available. I would also argue that we will need to make finer distinctions among the control states that range from reflexes to emotionsthe many functional features shown in figure 1 did not all appear at once, and some might disappear or reappear in certain species as adaptations to particular ecological pressures. None of this should be a surprise to biologists: the full set of features seen in humans will have partial sets across many other animal species that are piecemeal. The evolution of emotions is unlikely to have progressed with a rigid linear addition of features simply added to reflexes.

Nonetheless, it is worth speculating on the primacy and order of the emotion features listed in figure 1—and on ones that are possibly uniquely human. I consider priority over behaviour (a feature that Herbert Simon already stressed when he wrote of ‘interrupt mechanisms’; [45]) and valence (a feature that Charles Darwin had already stressed in his principle of antithesis; [1]) fundamental. Priority provides exactly the kind of automaticity and control by which emotions can mount behavioural responses more rapidly than mechanisms for goal-directed behaviour. If you had to think about what to do when faced with a charging tiger, you'd be eaten; your emotional state takes over and saves your life. Similarly, valence organizes emotional behaviours along their most basic axis: approach or withdrawal.

But priority and valence are evident already at the level of reflexes—they are the features that emotions inherit from reflexes. The next features in the list of figure 1 begin to distinguish emotions from reflexes. Generalization pertains to the fan-in/fan-out architecture that we have been discussing. Many different inputs can cause a state of fear: a tiger, a bear, a person with a gun, or merely the anticipation or hallucination of these. By contrast, the knee-jerk reflex can be caused only by a single kind of input. Similarly, my fear can cause me to quietly hide, run away or defend myself, whereas the knee-jerk reflex only moves the leg. Scalability is also absent for reflexes—they are ‘all or none’, whereas my fear can mount from anxiety to fear to panic (with different adaptive behaviours for each of these stages; [46]).

Now, which of the features we have reviewed so far are found in intermediate states, such as those that control mating rituals in birds? They evidence priority and valence. But they are not scalable, tending to be all-or-none. There is also relatively little generalization: specific sensory stimuli trigger the behaviours, and the behaviours are very stereotyped and not diverse. But, unlike reflexes and more like emotions, they do show complex, sequenced behaviours and the states evidence temporal persistence. So, I think it is likely that as we survey states from reflexes to emotions, we will find various intermediates, bird courtship rituals among them. Exactly where we decide to draw the line and classify states as true emotions may end up being more a matter of convention than scientific discovery.

The evolutionary elaboration of emotions from simpler reflex-like circuits on the one hand provides us with a scaffold for modelling emotion circuits, yet on the other hand it also shows us the complications that render our models inadequate. One such oversimplified principle is that a rapid, mostly feedforward circuit can trace sensory input to motor output (as in a reflex). While this may be at the core of emotional expression, it has been elaborated in important ways. One elaboration is that there is feedback everywhere, resulting in iterative, reverberative and persistent activity in the circuit (e.g. [47]). Aspects of these anatomical loops of processing mirror the more conceptual loops of iterative processing features in some leading psychological theories of emotion, such as the Component Process Model of appraisal theory [48]. These recursive themes in how an emotion unfolds highlight two important features: persistence and scalability. Emotions persist for some time, and they certainly continue throughout the temporally extended evaluation of a threat. Whereas the first loop of processing may trigger a defensive response, subsequent loops provide more information and may result in fleeing instead.

Related to the feedback elaborations just noted is the ability for cortical regions to ‘reach down’ into the subcortical circuits. This is a kind of ‘cognitive penetrability’ that violates the functional assumptions historically assigned to modular processing [49]. It is perhaps most obvious in the case of volitional regulation of emotion in adult humans, but it is seen even in simpler cases in other species. The ubiquitous feedback, as well as these more complex connections that skip hierarchical levels, violate many of the architectural features that artificial neural networks typically have. For instance, the type of sensory abstraction accomplished by central emotion structures like the amygdala or hypothalamus will differ from that possible with a standard feedforward deep convolutional neural network (for a clear example of how the brain deviates from such a model even for simpler object recognition, see [50]).

Another simplification in our models of how the brain accomplishes emotional expressions is the ‘fan-in/fan-out’ architecture that we have highlighted throughout: high-dimensional sensory input is compressed to a low-dimensional emotion representation, which in turn fans out to a high-dimensional coordination of emotion expression involving multiple effectors and complex, sequenced behaviours. It has been suggested that this is perhaps a rather general feature of nervous systems. For instance, the octopus takes high-dimensional sensory input about the visual appearance of its environment, compresses this to a low-dimensional central neural representation, and then is able to reconstruct the appearance of the environment on its skin—the amazing ability of camouflage that cephalopods have evolved (figure 3). I think that emotions face a similar challenge: the sensory input is high-dimensional and diverse (e.g. many different kinds of stimuli could be predators) and the behavioural output is high-dimensional (facial, musculoskeletal, autonomic and endocrine responses all need to be coordinated at the same time). But in the brain, we find neural representations that seem to code for a few basic dimensions of emotions—such as valence [19], or persistence and scalability [51]. Even the conscious experience of emotion (when assessed in humans with self-report ratings) seems to feature only three or four dimensions, regardless of whether the emotion is evoked by looking at images, at videos or from autobiographical recollection ([52]; for a summary of methods see [53]).

At the simplest level, such a scheme bears resemblance to statistical models that compress two high-dimensional datasets. Canonical correlation, for instance, imposes a linear optimization to link two high-dimensional datasets through low-dimensional modes. Perhaps most relevant are variational autoencoders, which accomplish more powerful nonlinear compression through symmetric encoding and decoding networks (this is also the mathematical analogy taken in figure 3 with respect to the octopus brain). These mathematical approaches mirror a fact about our world on which brains have also capitalized with ‘fan-in/fan-out’ architectures: while the world is complex and variable, it features plenty of correlations, allowing one to capture a lot of this variance with fewer dimensions more efficiently. Just take a look at the vast majority of entirely realistic-looking photos that typically come out of your camera: they are all JPEG compressed, summarizing their essence by eliminating redundancies.

One important and, in retrospect, obvious feature of the biological system that all the above-mentioned statistical models lack is an explicit incorporation of a causal mechanism. When we speak of ‘emotional expression’, we are implicitly assuming that the neural circuits discussed above are not merely associated with emotional behaviours, but cause them. Whereas methods like canonical correlation or autoencoders are symmetrical, the brain needs to go from sensory input to motor output, not the other way around. This causally asymmetric feature has, in fact, been incorporated into recent models—for instance, causal feature learning [54], which also aggregates high-dimensional datasets, but does so by explicitly modelling the causal relationships between them. Algorithm development for dimensionality reduction will be an important domain for future work, allowing us to better model how brains summarize relations between diverse stimuli and diverse behaviours through emotion states.

Finally, it should be noted that it is possible to eschew a causal mechanism altogether, at least one decomposable into a sequence of component steps, using alternative frameworks such as dynamical systems modelling. This has already been done for some of the components of an emotion mechanism, such as the description of a line attractor in the dynamical system model of population activity in the hypothalamus [44,51]. Another example is the behavioural state switching seen in the worm *Caenorhabditis elegans*, whose hunting behaviour has been studied in detail. Here, there are two hunting modes, and switching between them can be well modelled as a dynamical system [55]. In such models, the state space of neuronal activity is summarized by a few dimensions that account for most of the variance, and the time evolution of the system's state is a continuous trajectory in that state space. There are no discrete steps, and instead, the transformation of input to output is described by a smoothly varying differential equation. In general, such dynamical system models have been applied only to components of an emotion circuit (such as specific cell populations in the hypothalamus), but in principle, one could zoom out to include the entire brain, or even brain plus organism plus environment in such a model. This would stand in contrast to the anatomical partitioning of a mechanism that sequentially converts sensory input to motor output.

The picture that might emerge seems reminiscent of historical attempts to model complex biological processes with concepts like resonance or fields, or, perhaps most relevant here, ecologically structured relations between organism and environment [56]. Relatedly, the octopus's control of its high-dimensional arms arises from a complex interaction between nervous system control and the physical substrate—the body is literally participating in the computation of movement in these animals, a very different view of motor control from what we are used to in animals with hard body components (see [24], for review). In the same vein, it is important to emphasize that sensory and motor are not simply the termini of a linear causal chain: perception of emotions in both oneself and in their effect on others closes the loop. It is by now well known that there are ‘mirror neurons’ in the ventral premotor cortex of primates that both trigger complex actions and respond to the observation of that same action. Viewing facial expressions in humans elicits similar expressions in the viewer, and many of the circuits we discussed above have neurons that not only function to coordinate emotional expressions, but that also respond to the observation of those expressions.

I close with these considerations only to indicate that there are very different alternative models of emotions available in principle, and that emotions are embedded in a vastly complex causal web that includes both the social and non-social environment. But I don't propose endorsing these views as a useful starting point for a science of emotion. The difficulty with all of these more holistic schemes has been a clear advance in understanding and intervention. They may describe aspects of the phenomenon more completely, but that description is not as useful for explanation and manipulation. Given that the brain is in fact not equipotential, but consists of discrete structures with particular connectivity, we should instead attempt to understand the evolved functions that these components subserve. Anatomy matters, since it constrains and reflects function. What is missing is a time machine to help us see its evolution—and for that, we desperately need more comparative studies of emotions across species.

## Data Availability

This article has no additional data.

## Theme

One contribution of 19 to a theme issue ‘Mechanisms, development, phylogeny and functions of emotional expressions’.
